# Span of spleen is associated with disability status in multiple sclerosis: a cross-sectional abdominopelvic ultrasonography study

**DOI:** 10.1038/s41598-024-66216-5

**Published:** 2024-07-03

**Authors:** Masoud Etemadifar, Seyyed-Ali Alaei, Mehri Salari, Nahad Sedaghat

**Affiliations:** 1 Department of Neurosurgery, School of Medicine, Isfahan University of Medical Sciences, Isfahan, Iran; 2 Alzahra Research Institute, Alzahra University Hospital, Isfahan University of Medical Sciences, Isfahan, Iran; 3 Functional Neurosurgery Research Center, Shohada Tajrish Comprehensive Neurosurgical Center of Excellence, Shahid Beheshti University of Medical Sciences, Tehran, Iran; 4 Student Research Committee, Isfahan University of Medical Sciences, Isfahan, Iran; 5 Student Research Committee, Kashan University of Medical Sciences, Kashan, Iran; 6 School of Medicine, Isfahan University of Medical Sciences, Isfahan, Iran

**Keywords:** Multiple sclerosis, Liver, Multiple sclerosis, Predictive markers, Prognostic markers, Predictive markers, Prognostic markers

## Abstract

Characteristics of livers and spleens of people with multiple sclerosis (pwMS) could constitute good biomarkers of MS-related characteristics such as the disability status. To test the hypothesis “the gross anatomical features of livers and spleens, are not similar between pwMS with different disease characteristics” a cross-sectional study was conducted on pwMS seen at the Isfahan MS clinic, Iran, from February until December 2023. Definitive, otherwise-healthy, pwMS were enrolled after an initial laboratory evaluation. Presence/absence and grading of non-alcoholic fatty liver disease (NAFLD) and the span of spleen were determined by a radiologist using high-resolution abdominopelvic ultrasonography. 193 pwMS (160 women) were enrolled. Of whom, 143 (74.1%) were receiving first-line disease-modifying therapies (DMTs), 24 (12.4%) fingolimod, and 26 (13.5%) rituximab. The span of spleen was negatively associated with EDSS (adjusted β [SE] − 4.08 [1.52], *p* < 0.01), as well as 6 m-CDW (adjusted β [SE] − 6.94 [3.56], *p* = 0.05), unlike age, DMTs, and MS duration (all with *p* > 0.05). Receiver operating characteristic analysis showed, spleen span performs significant but poor in discrimination of EDSS > 1 from EDSS = 1 (area under curve [AUC] 0.62, SE 0.05, *p* < 0.01), yet, significant and fair in discrimination of presence from absence of 6 m-CDW (AUC 0.72, SE 0.06, *p* < 0.01). Other findings were unremarkable. Further longitudinal, prospective studies are warranted to confirm whether smaller spleens are predictive of higher disability accrual rate in pwMS. Particularly, findings require further validation in untreated/treatment-naïve pwMS, and ones with higher EDSS scores.

## Introduction

Multiple sclerosis (MS) is one of the demyelinating diseases of the CNS, the etiology of which remains unknown. A wide variety of factors, including immune dysregulation, viral infections, metabolic disturbances, and systemic inflammation, have been shown to be associated with pathogenesis, and/or progression of MS. Such factors may impact, or may be impacted, by other organs as well. For instance, Melero-Jerez et al. showed in a murine model, that the proportion of myeloid-derived suppressor cells (MDSCs) in the spleen, is associated with the severity of the clinical course and extent tissue damage in CNS demyelination^1^. Thereby, the features of spleen might be influenced, hence, the people with more severe MS may have spleens of different features than the others. Furthermore, viral infections have also been implicated in the development and exacerbation of MS. Epstein-Barr virus (EBV) is one such virus that has been extensively studied in relation to its association with MS^2,3^. EBV infection is known to impact multiple abdominal organs, and is known to be associated with changes in anatomical features thereof, especially the spleen^4^.

In addition, metabolic disturbances, including non-alcoholic fatty liver disease (NAFLD) – which is diagnosed based on changes in anatomical features of the liver^5^—and the onset/severity of MS, could be linked via their underlying factors. For instance, vitamin D deficiency and dyslipidemia, could be associated with NAFLD as well as the MS course, and the response thereof to treatment^6,7^. In line with that, Tremlett et al*.* have shown, pwMS bear an increased relative risk (RR) of liver test abnormalities, and particularly, elevated aminotransferases^8^, the most common cause of which is known to be NAFLD^9,10^. Moreover, pwMS are often treated with a wide variety of medications; these could impact their organs as well.

The anatomical features of the abdominopelvic organs like the liver and the spleen, e.g., their span, morphology, and composition, are features that could be easily measured in clinical settings e.g., using abdominopelvic ultrasonography (APUS). Given this, and that many of the pathophysiological, and pharmacological pathways that are involved in pwMS, whether known or unknown, could impact the anatomical features of these organs as well, these features may be valuable indirect biomarkers of the disease course and/or safety/effectiveness of treatments. In other words, the MS pathology may not be directly associated with abdominopelvic organs, yet, many underlying factors are present that could link the MS severity with abdominopelvic organs, rendering those particularly good, indirect biomarkers of the disease activity. Yet, to date, and to the best of our knowledge, these features have not been explored for such utilities in pwMS.

Apart from their potential as indirect biomarkers, the association between the anatomical features of abdominopelvic organs and MS, could help elucidate the complex interplay between viral agents, metabolic disturbances, immune dysregulation, systemic manifestations, and MS pathogenesis. Understanding these relationships could contribute to the development of targeted interventions and personalized treatment strategies for pwMS.

Therefore, we aimed to study the anatomical features of abdominopelvic organs, and their association with different characteristics of MS. We hypothesized “the anatomical features of livers and spleens, are not similar between pwMS with different disease characteristics.” Hereby, our study is reported in accordance with the strengthening the reporting of observational studies in epidemiology (STROBE) statement.

## Methods

### Study design and setting

The study presented herein, was an observational, cross-sectional study, conducted from February until December 2023, on definitive, otherwise-healthy pwMS (further described infra in Section "Participants"), who were seen at the Isfahan MS clinic, Iran. After obtaining of informed consent, the potential participants were evaluated by a neurologist, their baseline information was extracted from their electronic medical records (EMRs) at the clinic, and they were referred for blood tests to determine their eligibility. Thereafter, they were referred to a pre-specified radiologist, who determined the anatomical features of their livers and spleens, by means of APUS. Furthermore, characteristics of other abdominopelvic organs, such as the kidneys and the urinary tract, and the biliary system, were explored as well.

### Participants

The following were the inclusion criteria for the participants, along their rationale:

1. Being able, and willing to provide a written informed consent; to ensure that participants have a clear understanding of the study's purpose, procedures, risks, and benefits, and acknowledge their voluntary agreement to participate;
2. Being at least, 18 years of age; to ensure that they have the legal capacity to make informed decisions, while representing the broader population of adults with MS;
3. Definitive diagnosis of MS, made by a neurologist, based on the 2017 McDonald criteria^11^, at least, a year prior to recruitment; to minimize the risk of misdiagnosis or inclusion of individuals with mimic neurological conditions (e.g., other CNS demyelinating diseases), and since precise specification of MS phenotype (relapsing or progressive) requires at least 1 year of clinical follow-up^11^.
4. Being in a neurologically-stable or improving state, defined as experiencing no new neurological abnormality, or worsening of a pre-existing one, in the last 30 days; as instability of neurological symptoms may have confounded study findings;
5. Having no history of significant neurological conditions (e.g., Parkinson's disease, stroke) that may confound the assessment of MS prognosis; to help isolate the effect of MS and associated treatments on the outcomes being measured;
6. Having no history of significant comorbidities that may impact APUS findings or MS prognosis (e.g., diabetes mellitus [DM], morbid obesity, alcoholism, advanced liver disease, end-stage renal failure, hematologic malignancy, etc.); to minimize the influence of confounding factors on the outcomes being measured;
7. Being non-pregnant and non-breastfeeding; as several physiological parameters undergo changes during pregnancy and breastfeeding, which could have affected the outcomes being measured; and
8. Having the ability to undergo APUS, while having no contraindications therefor (e.g., severe claustrophobia); as participants must have been able to undergo APUS safely.

Furthermore, the following exclusion criteria were defined:

1. Withdrawal of informed consent at any time; and
2. Diagnosis of significant neurological conditions, and/or significant comorbidities, and/or pregnancy, as defined above, and/or hospitalization and/or death, until one month after the initial evaluation.

With the above criteria in mind, a specified neurologist identified potential participants, from the pwMS that were being seen for their routine follow-up, obtained written informed consent, and following a comprehensive clinical assessment as done in routine follow-up visits, referred them for blood tests to ensure their eligibility, wherein the blood tests included complete blood count with differentials (CBC diff), hemoglobin (Hb) level, fasting blood glucose, glycated hemoglobin (HbA1C) level, blood urea nitrogen (BUN) and creatinine (Cr) levels, liver enzyme tests, total and conjugated bilirubin levels, albumin level, prothrombin time with international normalized ratio (PT/INR), partial thromboplastin time (PTT), and lipid panel. The blood tests were omitted, if the potential participant had them performed in the prior year, in which case, the results of those were retrieved and used. It should be noted that these measures were among neither our exposure nor outcome variables, as the samples were not taken on the same day as the APUS, and analyzed in different laboratories, despite them being accredited and compliant with good laboratory practices, and holding the latest national standard certifications which require undertaking and passing regular tests comprising inter-laboratory comparisons, to ensure provision of accurate results. We considered such results adequate only as auxiliary means to the clinical assessments, for ensuring absence of chronic conditions that may impact the outcomes (as defined supra in the inclusion criteria).

### Variables and their measurement

The primary outcome variable of the study was defined as the presence/absence and grade of NAFLD (no NAFLD, grade I, grade II, grade III). The secondary outcome variable of the study was defined as the longitudinal span of spleen. Both of these variables were measured via high-resolution APUS by an experienced radiologist. The grading of NAFLD was defined as follows; no NAFLD: normal liver echogenicity; grade I: presence of a diffuse increase in liver echogenicity, with sparing of periportal and diaphragmatic regions; grade II: presence of a diffuse increase in liver echogenicity, involving periportal regions, with sparing of diaphragmatic region; and grade III: presence of a diffuse increase in liver echogenicity, involving periportal and diaphragmatic regions. The longitudinal span of spleen was measured in millimeters. Further exploratory outcome variables included presence/absence of morphological abnormality of liver and/or spleen, length of kidneys, presence/absence of biliary and/or urinary system stones, and presence/absence of any other abnormality in the APUS studies.

Furthermore, the baseline variables, serving as potential confounders, were age, sex, duration of MS, and the current DMT; these were obtained from EMRs. The current expanded disability status scale (EDSS) score, as determined by the recruiting neurologist through neurological examination, served as the primary predictor variable. As mentioned supra, being in a stable neurological state served as an inclusion criterion, hence, EDSS measurements were done when participants were neurologically stable. Another independent variable included the presence/absence of 6-month confirmed disability worsening (6 m-CDW) on the EDSS, in one year prior to recruitment; data on this variable was obtained in a retrospective fashion from EMRs.

### Bias

The APUS studies are known to be subjective, and operator-dependent, hence, leading to potential bias in research settings. In order to address this problem, only a single radiologist, with a significant experience in APUS, was pre-determined for all the APUS studies. Furthermore, to address measurement bias, the radiologist was not involved in the study, and therefore, was blind to all data except for the demographics of participants, prior to performing the measurements.

### Study size

An accurate sample size calculation was not possible, since prior studies reporting the primary outcome measures, were not found, to enable anticipation of effect sizes and precision measures. Nevertheless, since NAFLD is the most common cause of abnormal liver enzyme test results, we used a prior study on liver enzyme tests of pwMS^8^ to estimate a sample size, whereby, considering an alpha value of 0.05 and a beta value of 0.2, a minimum sample size of 199 people was estimated.

### Statistical analysis

Descriptive data pertaining to all variables were presented using appropriate aggregate, along confidence values, such as mean and standard error (SE) or standard deviation (SD) values, median and range values, count and percentage values, etc. Missing values were planned to be handled as follows, to maximize the usage from the data: wherein they pertained to baseline and/or predictor variables, they were planned to be imputed using an automated imputation strategy with 10 imputations; wherein they pertained to outcome variables, they were planned to be excluded from the analysis.

Appropriate (non)parametric statistical tests were used to test the study hypotheses. Furthermore, univariate generalized linear models (UGLM) were used for unadjusted analyses, and multivariable generalized linear models (MGLM) were used to investigate the effect of predictor variables on the outcome variables, as well as accounting for the potential confounding variables. The results of UGLM and MGLMs were reported using unadjusted β and adjusted β (aβ), respectively, along SE, and *p* values. Furthermore, a receiver operating characteristic (ROC) analysis was performed to assess the APUS findings as biomarkers of the disability status measures, such as EDSS score and 6 m-CDW. The results of the ROC analyses were reported using area under curve (AUC), SE, and *p* values. AUC values were further stratified based on previous literature^12^ and used to qualitatively describe the performance of the investigated variables as follows: 0.5–0.6, fail; 0.6–0.7, poor; 0.7–0.8, fair; 0.8–0.9, good; and 0.9–1, excellent. The criterion of rejecting the null hypothesis was determined as a *p* value equal or below 0.05. The software used for means of statistical analysis and constructing graphic representation of results, comprised SPSS statistics (version 23, IBM Inc.) and Prism (version 9, GraphPad software Inc.)

### Ethics

The present study was conducted in accordance with the declaration of Helsinki. All participants within the present study provided a written informed consent to participate. The present study was approved by the research ethics committee (REC) of the Shahid Beheshti University of Medical Sciences.

## Results

### Participants

**Figure 1:**
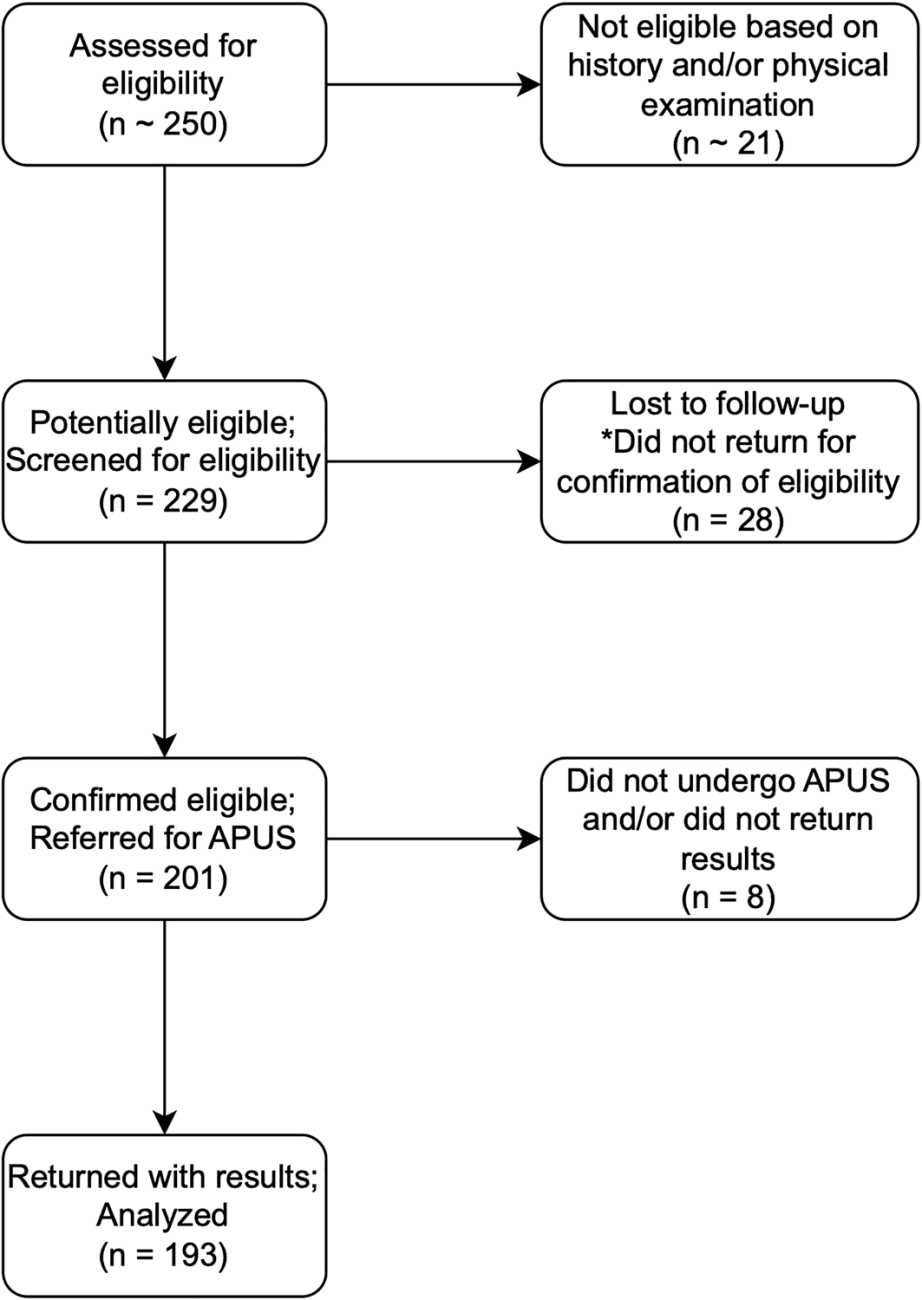
Study flow diagram. Abbreviation: APUS, abdominopelvic ultrasonography.

Figure 1 shows the flow diagram of the study. Around 250 pwMS were screened for eligibility. 229 pwMS were considered potentially eligible; all of whom were referred to be further examined for eligibility by blood tests. Of whom, 28 never returned and did not respond to follow-up calls, while 201 (87.7%) returned, and were confirmed to be eligible, and were referred for APUS. Of whom, 193 (96%) underwent APUS by the time of our final follow-up, and were included in the study.

### Descriptive data

Table 1 summarizes the baseline characteristics of the 193 included participants. Data pertaining to the primary outcome, i.e., the presence/absence and grading of NAFLD, was available for all included participants. Quantitative data pertaining to the secondary outcome, i.e., the span of spleen, was available for 149 (77.2%) of the included participants; the remaining were reported as “within normal limits” without precise quantification. As interpreted from Table 1, participants with available quantitative data on span of spleen were similar in characteristics to the overall population. Furthermore, data pertaining to the length, and parenchymal thickness of kidneys, was available for 121 (62.7%), and 84 (43.5%) of the included participants, respectively; these were similar in characteristics to the overall population as well. No imputations were done, since no data was missing regarding predictor variables.

**Table 1:** Characteristics of participants.

Variable	All participants (n = 193)	Participants with available span of spleen (n = 149)
Age (mean, SD) [years]	40.7 (10.1)	39.7 (10.5)
Sex (n of women, %)	160 (82.9)	129 (86.6)
Duration since MS onset (mean, SD) [years]	10.1 (7.6)	10 (7.5)
Subtype of MS (n, %)		
Relapsing remitting	181 (93.8)	140 (94)
Secondary progressive	12 (6.2)	9 (6)
DMT (n, %)		
Interferons	46 (23.8)	35 (23.5)
Glatiramer acetate	17 (8.8)	12 (8.1)
Dimethyl fumarate	48 (24.9)	38 (25.5)
Teriflunomide	32 (16.6)	24 (16.1)
Fingolimod	24 (12.4)	17 (11.4)
Rituximab	26 (13.5)	23 (15.5)
Current EDSS score (median, range)	1 (4)	1 (4)
6 m-CDW in prior year (n, %)	24 (12.4)	20 (13.4)
Elevated ALT* (n, %)	61 (31.6)	45 (30.2)
NAFLD status (n, %)		
No NAFLD	132 (68.4)	103 (69.1)
Grade I	52 (26.9)	40 (26.8)
Grade II	9 (4.7)	6 (4)
Span of spleen (mean, SD) [mm]	NA	98.3 (13.7)

SD, standard deviation; MS, multiple sclerosis; DMT, disease-modifying therapy; EDSS, expanded disability status scale; 6 m-CDW, 6-month confirmed disability worsening; ALT, alanine aminotransferase; NAFLD, non-alcoholic fatty liver disease; mm, millimeters. *As defined by the American College of Gastroenterology^15^.

Moreover, no morphological abnormality was detected, except for an accessory spleen spanning 9 mm adjacent to the main spleen, in a 29-year-old woman with relapsing remitting MS on interferon therapy. Additionally, the following constituted incidental findings: simple cysts, and angiomyolipoma of the kidney in 7 (3.6%) and 2 (1%) of the participants, respectively; kidney stones with cumulative size of less than 10 mm and without hydroureteronephrosis in 12 (6.2%) of the participants; gallbladder sludge or stones with cumulative size of less than 20 mm in 8 (4.1%) of the participants; gallbladder polyp and increased gallbladder size suggestive of chronic cholecystitis in 3 (1.6%) and 2 (1%) of the participants, respectively; liver and spleen hemangioma in 7 (3.6%) and 1 (0.5%) of the participants, respectively; a liver hydatid cyst in 1 (0.5%) of the participants; and urinary bladder prolapse with a 68 mm uterine leiomyoma in a single participant. None of these findings were associated with any symptoms.

### Outcome data

Outcome data are summarized in Table 1. As interpreted, 52 (26.9%), 9 (4.7%), and no participants had grade I, grade II, and grade III NAFLD, respectively; the crude prevalence of NAFLD for both women (RR [95% confidence interval (CI)] 1.04 [0.77, 1.39], *p* = 0.74) and men (RR [95% CI] 0.99 [0.49, 1.77], *p* = 0.99) with MS as calculated in the present study were comparable to that reported for the general Iranian population in a recent meta-analysis involving more than 30 thousand adults^13^. An older meta-analysis study^14^ reported similar values as well. Nevertheless, caution is advised in interpretation of this comparison, as different diagnostic tools may have been utilized in the present study and the ones included in the meta-analysis^13^. Furthermore, based on the values put forth by the American College of Gastroenterology (ACG)^15^, 47/160 (29.4%) of the women, and 14/33 (42.4%) of the men (61/193 [31.6%] of the total pwMS) had elevated ALT levels, of whom, 17 (27.9%) had grade I, and 4 (6.5%) had grade II NAFLD (Pearson Chi^2^: 0.82, *p* = 0.66). In a recent cross-sectional study done on more than five thousand general adults of similar ethnicity and socioeconomic status with our study, prevalence of elevated ALT based on ACG values has been reported to be 17.7% in women and 27.3% in men^16^; hence, in our study, both the women with MS (RR [95% CI] 1.66 [1.29, 2.14], *p* < 0.001) and the men with MS (RR [95% CI] 1.56 [1.04, 2.33], *p* = 0.03) had an increased risk of having elevated ALT levels.

**Figure 2:**
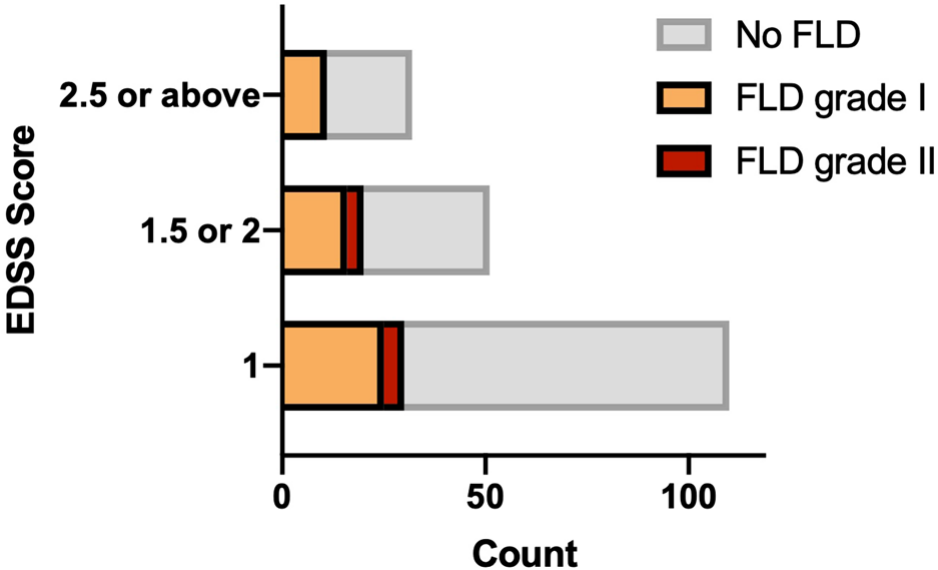
Status of NAFLD among participants as determined through APUS, stratified by EDSS score. Abbreviations: APUS, abdominopelvic ultrasonography; EDSS, expanded disability status scale; FLD, non-alcoholic fatty liver disease.

**Figure 3:**
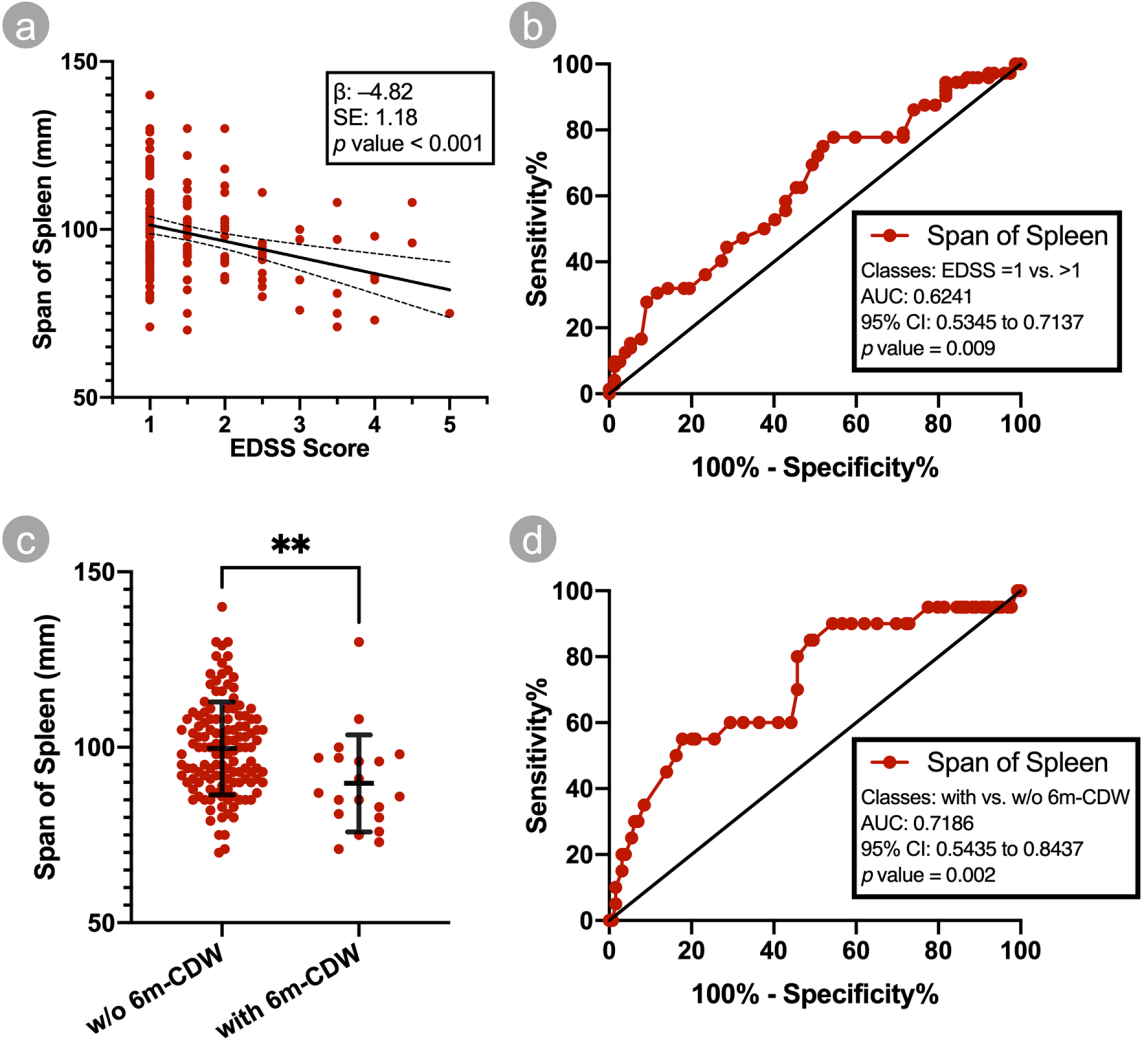
(**a**) scatter plot of span of spleen against EDSS score; the bold and hyphenated lines correspond to the regression and 95% confidence interval lines, respectively. (**b**) ROC curve of span of spleen for discrimination of EDSS = 1 from > 1. (**c**) scatter plot of span of spleen against presence or absence of 6 m-CDW in the prior year; the horizontal bold lines correspond to mean and standard deviation values and the vertical bold lines correspond to the values within one standard deviation of the mean. (**d**) ROC curve of span of spleen for discrimination between presence and absence of 6 m-CDW in the prior year. Abbreviations: mm, millimeters; EDSS, expanded disability status scale; SE, standard error; AUC, area under curve; CI, confidence interval; w/o, without; 6 m-CDW, 6-month confirmed disability worsening; **p < 0.01.

With (non)parametric hypothesis testing, no significant difference was confirmed in the presence/absence of NAFLD in pwMS with different EDSS scores (Pearson Chi^2^ 11.84, *p* = 0.16) (Fig. 2), and with/without 6 m-CDW (Pearson Chi^2^ 1.28, *p* = 0.26). Furthermore, no significant difference in EDSS scores was confirmed between pwMS with grade I or grade II NAFLD (Mann–Whitney U 192.5, *p* = 0.36). Moreover, a significant difference was confirmed regarding the span of spleen in pwMS with different EDSS scores (F 2.41, *p* = 0.02), and with/without 6 m-CDW (mean difference [SD] 9.92 [3.20], *p* = 0.002) (Fig. 3c). Regarding the length and cortical thickness of the right and left kidneys, no difference could be confirmed in pwMS with different EDSS scores and/or with/without 6 m-CDW (all *p* values above 0.05).

Ordinal logistic UGLM and MGLM were utilized to further investigate the presence/absence and grading of NAFLD in pwMS with different age, sex, disease duration, DMT, and EDSS scores or 6 m-CDW (ordinal scale response; 0, no NAFLD; 1, grade I NAFLD; 2, grade II NAFLD). As it is depicted in Tables 2, 3, and 4, significant trends were identified through univariate models, for age and dimethyl fumarate therapy; the only significant trend in NAFLD status, confirmed through multivariable models, pertained to age. Of note, regarding EDSS scores, a significant trend was confirmed neither with the UGLM (β 0.10, SE 0.14, *p* = 0.56) nor with the MGLM (aβ − 0.02, SE 0.23, *p* = 0.91).

**Table 2:** Results of univariate generalized linear models.

Predictor	NAFLD (no NAFLD: 0; grade I NAFLD: 1; grade II NAFLD: 2) [ordinal logistic] (β [SE], *p* value)	Span of spleen (mm) [linear] (β [SE], *p* value)
Age	**0.05 (0.02), < 0.01**	**− 0.25 (0.10), 0.02**
Sex		
Female	(ref)*	(ref)*
Male	0.11 (0.40), 0.78	**7.98 (3.21), 0.01**
MS duration	0.02 (0.02), 0.35	**− 0.35 (0.15), 0.02**
DMT		
IFN-β	(ref)*	(ref)*
GA	–0.70 (0.65), 0.28	3.51 (4.42), 0.43
DMF	**–0.96 (0.46), 0.04**	1.49 (3.09), 0.63
TERI	–0.15 (0.46), 0.75	–2.20 (3.50), 0.53
FINGO	–0.59 (0.53), 0.27	**− 7.80 (3.90), 0.05**
RTX	–0.36 (0.50), 0.48	**− **5.35 (3.54), 0.13
EDSS score	0.10 (0.17), 0.56	**− 4.82 (1.18), < 0.01**
6 m-CDW		
No	(ref)*	(ref)*
Yes	0.53 (0.44), 0.23	**− 9.92 (3.18), < 0.01**

NAFLD, non-alcoholic fatty liver disease; SE, standard error; mm, millimeters; ref, reference; MS, multiple sclerosis; DMT, disease-modifying therapy; IFN-β, interferon-β; GA, glatiramer acetate; DMF, dimethyl fumarate; TERI, teriflunomide; FINGO, fingolimod; RTX, rituximab; EDSS, expanded disability status scale; 6 m-CDW, 6-month confirmed disability worsening. * The coefficients for the other categories are compared to this “ref” category.

Results with p value equal or below 0.05 are bolded.

**Table 3:** Results of multivariable generalized linear models involving EDSS score; results with p value equal or below 0.05 are bolded.

Predictor	NAFLD (no NAFLD: 0; grade I NAFLD: 1; grade II NAFLD: 2) [ordinal logistic] (aβ [SE], *p* value)	Span of spleen (mm) [linear] (aβ [SE], *p* value)
Age	**0.05 (0.02), 0.01**	–0.10 (0.12), 0.42
Sex		
Female	(ref)*	(ref)*
Male	0.06 (0.43), 0.89	**7.61 (3.07), 0.01**
MS duration	–0.01 (0.03), 0.68	0.001 (0.17), 0.99
DMT		
IFN-β	(ref)*	(ref)*
GA	–0.33 (0.68), 0.62	2.37 (4.35), 0.59
DMF	–0.63 (0.49), 0.19	0.29 (3.07), 0.92
TERI	–0.28 (0.49), 0.57	**− **1.86 (3.43), 0.59
FINGO	–0.40 (0.57), 0.48	**− **5.95 (3.80), 0.12
RTX	–0.22 (0.58), 0.70	0.86 (3.88), 0.82
EDSS score	–0.02 (0.23), 0.91	**− 4.08 (1.52), < 0.01**

NAFLD, non-alcoholic fatty liver disease; SE, standard error; aβ, adjusted β; mm, millimeters; ref, reference; MS, multiple sclerosis; DMT, disease-modifying therapy; IFN-β, interferon-β; GA, glatiramer acetate; DMF, dimethyl fumarate; TERI, teriflunomide; FINGO, fingolimod; RTX, rituximab; EDSS, expanded disability status scale. * The coefficients for the other categories are compared to this “ref” category.

**Table 4:** Results of multivariable generalized linear models involving 6 m-CDW; results with p value equal or below 0.05 are bolded.

Predictor	NAFLD (no NAFLD: 0; grade I NAFLD: 1; grade II NAFLD: 2) [ordinal logistic] (aβ [SE], *p* value)	Span of spleen (mm) [linear] (aβ [SE], *p* value)
Age	**0.05 (0.02), 0.01**	**− **0.17 (0.12), 0.17
Sex	0.10 (0.43), 0.81	
Female	(ref)*	(ref)*
Male	0.10 (0.43), 0.81	**7.44 (3.12), 0.02**
MS duration	–0.02 (0.03) 0.46	**− **0.03 (0.17), 0.86
DMT		
IFN-β	(ref)*	(ref)*
GA	–0.34 (0.68), 0.61	2.53 (4.39), 0.56
DMF	–0.67 (0.49), 0.17	**− **0.34 (3.10), 0.91
TERI	–0.27 (0.49), 0.58	**− **1.92 (3.47), 0.56
FINGO	–0.58 (0.57), 0.31	**− **7.09 (3.79), 0.06
RTX	–0.52 (0.56), 0.35	**− **1.68 (3.66), 0.65
6 m-CDW		
No	(ref)*	(ref)*
Yes	0.68 (0.54), 0.21	**− 6.94 (3.56), 0.05**

NAFLD, non-alcoholic fatty liver disease; SE, standard error; aβ, adjusted β; mm, millimeters; ref, reference; MS, multiple sclerosis; DMT, disease-modifying therapy; IFN-β, interferon-β; GA, glatiramer acetate; DMF, dimethyl fumarate; TERI, teriflunomide; FINGO, fingolimod; RTX, rituximab; 6 m-CDW, 6-month confirmed disability worsening. * The coefficients for the other categories are compared to this “ref” category.

Linear UGLM and MGLM were utilized to further investigate the span of spleen in pwMS with different age, sex, disease duration, DMT, and EDSS scores or 6 m-CDW. As it is depicted in Tables 2, 3, and 4, significant trends were identified in univariate models, regarding age, sex, disease duration, fingolimod therapy, EDSS score and 6 m-CDW. Of which, only the ones pertaining to sex, EDSS score, and 6 m-CDW were maintained in the multivariable models. This suggests that the significant trends seen in univariate models, pertaining to age, disease duration, and fingolimod therapy, are probably due to the association of those with disability status. Further of note, higher EDSS scores were associated with smaller spans of spleen, as confirmed by the UGLM (β − 4.82, SE 1.18, *p* < 0.001)(Fig. 3a) and the MGLM (aβ − 4.09, SE 1.52, *p* = 0.007) which hints towards an independent association; presence of 6 m-CDW was associated as well, with smaller spans of spleen, as confirmed by the UGLM (β − 9.91, SE 3.18, *p* = 0.002) and the MGLM (aβ − 6.94, SE 3.55, *p* = 0.05).

A ROC analysis was done to investigate the potential value of span of spleen, as a biomarker of EDSS = 1 versus EDSS > 1, and/or presence/absence of 6 m-CDW. Results showed that the performance of span of spleen for discrimination of EDSS = 1 versus EDSS > 1 is significant, yet, poor by itself (AUC 0.62, SE 0.05, *p* = 0.009) (Fig. 3b). The span of spleen performed significant and fair in discrimination between presence or absence of 6 m-CDW (AUC 0.72, SE 0.06, *p* = 0.002)(Fig. 3d).

Further analyses were done to investigate the association of the variables mentioned supra, with the length and cortical thickness of the left and right kidneys. All results pertaining to these analyses returned unremarkable (results not shown).

## Discussion

In a cross-sectional fashion, we investigated the findings of APUS studies in pwMS with different characteristics, with a particular focus on the current disability status on the EDSS. Our remarkable findings comprised an increased risk of elevated ALT in the pwMS, relative to the general adult population, and a significant negative association between the disability measures and the span of spleen, which was independent from age, sex, disease duration, and DMT. We further showed with ROC analysis, the span of spleen constitutes a significant but suboptimal imaging biomarker of the disability status in pwMS, the value of which, particularly in conjunction with other prognostic markers, merits further investigation.

It could be argued, the findings of APUS as seen in pwMS are limited in value as biomarkers, since they are present in a considerable number of general people, and as various confounding factors such as genetic factors, dietary habits, other etiologies of hepatitis, alcohol/painkiller consumption, etc., exist that limit the attributability of APUS findings to MS. In this regard, it should be noted that that (i) the extent of presence of a factor in general population may be independent from its prognostic – rather than diagnostic—value, e.g., a considerable proportion of the general population are men, and smoke, yet, male sex and smoking are known prognostic factors in MS^17,18^; and (ii) although not directly attributable to MS pathogenesis, many underlying factors e.g., genetic factors, obesity^19^, smoking^17^, dyslipidemia^20^, etc., could link abdominopelvic organs—therefore, APUS findings—to MS prognosis; hence, APUS findings could constitute indirect—rather than direct—biomarkers of MS prognosis, regardless of the underlying factors that link them together. It should also be emphasized that such links do not necessarily mean causation.

In a prior study, Tremlett and colleagues found that pwMS treated with placebo bear an increased risk of presenting with elevated ALT levels^8^, whereby the etiology was not investigated. In line therewith, our study, done on pwMS mostly treated with first-line DMTs, also showed an increased risk of elevated ALT in pwMS, yet, the amount of which was lower in our study (RR around 1.5 in our study vs. 3.7 as reported by Tremlett et al.). Furthermore, although the most common etiology underlying elevated ALT has been reported to be NAFLD^9,10^, in our study, APUS revealed NAFLD only in around a third of the pwMS with elevated ALT. The etiology behind the elevated ALT in the other two-thirds of the pwMS remains to be investigated; inadequate sensitivity of APUS, hepatotoxicity of DMTs, infectious etiologies, including viral ones, unidentified ingestion of hepatotoxic materials, including drugs, and autoimmune processes, are among the possible explanations. Also of note, two thirds of the pwMS with NAFLD did not have elevated ALT levels, which is consistent with studies among general population^21^.

Moreover, we found that smaller spleens, higher EDSS scores, and presence of 6 m-CDW, are associated with one another; the reason is unknown since this subject is understudied. In this regard, a prior murine study is of note; Tsunoda and colleagues found that experimental allergic encephalomyelitis (EAE) mice with greater disability accrual rates bear atrophic spleens, as well as other lymphoid organs^22^. They further demonstrated this atrophy to be secondary to “massive” apoptosis of the lymphocytes within these organs. Whether this is the case with MS as well, the underlying mechanism of such apoptosis, and whether EBV, a lymphotropic entity strongly associated with MS, plays a role in this regard, remains to be studied. Chronic inflammation, and recurrent obstructions in capillary beds – such as the phenomenon seen in sickle cell disease, whether or not associated with EBV, constitute alternative hypotheses that remain to be investigated. In addition, the span of spleen may be differently affected by DMTs used for pwMS with lower or higher disability accrual rates, which may explain this observation. This is of note, since it has been shown that DMTs such as fingolimod^23–25^, dimethyl fumarate^26^, and teriflunomide^27^ impact the cells residing within the spleen; our study, however, does support this explanation. Our univariate models initially demonstrated an association only between fingolimod and the span of spleen, yet, this association was not retained in multivariable models that involved disability accrual measures, suggesting that the association between disability accrual measures and the span of spleen is independent from DMTs. Nevertheless, our study had an important limitation in this regard, lacking untreated pwMS as reference; therefore, this remains an interesting subject for future investigations.

Furthermore, in ROC analysis, the span of spleen performed poor in discrimination of EDSS > 1 from EDSS = 1, but fair in discrimination of presence from absence of 6 m-CDW; this, which is also reflected in regression coefficients, suggests that the association is more pronounced with more recent disability accrual. Nevertheless, these results suggest that span of spleen does not perform optimal as a sole prognostic biomarker of disability accrual in clinical settings, yet, the significance of hypothesis testing—i.e., the low *p* values—indicates span of spleen could be of additional value in conjunction with other markers rather than a sole biomarker; this also remains to be investigated in future studies.

## Limitations

Last but not least, our study has been observational and cross-sectional, and bears all limitations associated with such studies. Further of note, some risk factors associated with APUS findings, especially body mass index, and some aspects of MS, e.g., relapse activity and MRI measures, have not been considered in our study.

Furthermore, no untreated pwMS were included in our study as participants due to lack of treatment-related eligibility criteria, their paucity at our center, and that they generally bear more at risk for metabolic comorbidities due to advanced age and/or advanced disease, or have halted treatment for pregnancy and/or breastfeeding-related reasons, hence, failing to meet the eligibility criteria. Additionally, our third inclusion criterion prevented inclusion of treatment-naïve participants (upon diagnosis). This is acknowledged as a potential source of bias. Future studies are warranted for validation of findings in this subset of pwMS. Moreover, no eligibility criteria were set based on EDSS score, hence, the distribution of the EDSS scores of the study participants simply reflects the distribution of the EDSS scores of the pwMS seen at our center. It should be appreciated that having more participants with higher EDSS scores would have added value to the results. Further studies are required to validate findings in pwMS with higher EDSS scores.

Also, although it was deemed unlikely for asymptomatic persons with normal blood test measures within a year to bear any acute and/or chronic condition that could impact the ultrasound measures significantly, it should be acknowledged that that taking the blood samples from the participants on the same day of the APUS and having them analyzed at a single laboratory would have been a more robust course of action, and would have been particularly of value for exclusion of acute conditions that might have been present.

Additionally, although the participants were referred to the radiologist with a detailed set of requests comprising a request for quantification of the span of spleen, the radiologist reported this measure to be “within normal limits” without mentioning a precise value in some of the cases, therefore, these cases could not be included in the analyses. Since this measure was a secondary outcome, the characteristics of people with precise quantification resembled the overall population, and the statistical analyses and hypotheses testing indicated adequate statistical power at the end, we did not refer the participants for a second time for the precise quantification of the span of spleen, yet, the potential impact of this on the results should be acknowledged and further investigated in future studies.

With all that in mind, further studies are warranted, especially ones that are longitudinal and prospective in design. The results of the present study should currently be interpreted with caution until results of further studies become available.

## Conclusion

Our study revealed (i) an increased risk of elevated ALT in pwMS relative to general population, only a third of which could be explained by NAFLD; (ii) the rate of elevated ALT in pwMS with NAFLD is comparable to that of general population; and (iii) a negative association between measures of disability accrual and span of spleen, which was independent from age, sex, MS duration and DMTs, and the underlying reason for which remains to be investigated. Furthermore, presence/absence and grading of NAFLD, length and cortical thickness of kidneys, were associated with neither disability accrual measures, nor DMTs and MS duration. Additionally, it should be noted the findings of the present study e.g., the proportion of participants with elevated ALT and/or NAFLD and/or the association of span of spleen with disability accrual measures do not necessarily imply any temporal and/or causal relationship(s), but point towards the merit of further, longitudinal studies on the subject. Some limitations are present in our study; hence, further studies, particularly longitudinal, prospective ones, are warranted, and results should be interpreted with caution. Of note, findings are especially not generalizable to the subset of treatment-naïve and/or untreated pwMS, as such pwMS were not included in the study.

## Data Availability

Data are available from the corresponding author for eligible researchers upon reasonable request and subject to approval of the REC.
